# Dermatologic presentations of hyper IgE syndrome in pediatric patients

**DOI:** 10.1186/s13223-025-00963-6

**Published:** 2025-05-02

**Authors:** Mohammad Mahjoubi, Ronak Rashedi, Noosha Samieefar, Fahimeh Abdollahimajd, Nima Rezaei

**Affiliations:** 1 Network of Interdisciplinarity in Neonates and Infants (NINI), Universal Scientific Education and Research Network (USERN), Tehran, Iran; 2 Clinical Research Development Center, Najafabad Branch, Islamic Azad University, Najafabad, Iran; 3 Pediatric Chronic Kidney Disease Research Center, Gene, Cell & Tissue Research Institute, Children’s Medical Center, Tehran University of Medical Sciences, Tehran, Iran; 4 Clinical Research Development Unit, Shohada-e Tajrish Hospital, Shahid Beheshti University of Medical Sciences, Tehran, Iran; 5 Research Center of Artificial Intelligence in Health, Shohada-e Tajrish Hospital, Shahid Beheshti University of Medical Sciences, Tehran, Iran; 6 Research Center for Immunodeficiencies, Children’s Medical Center, , Tehran University of Medical Sciences, Tehran, Iran; 7 Department of Immunology, School of Medicine, Tehran University of Medical Sciences, Tehran, Iran

**Keywords:** Job syndrome, Skin manifestations, Primary immunodeficiency diseases

## Abstract

**Background:**

Hyper-IgE Syndrome, also known as Job’s syndrome, is a rare primary immunodeficiency disorder characterized by recurrent infections and elevated levels of immunoglobulin E. While respiratory and systemic manifestations have been more emphasized, dermatological manifestations in Hyper-IgE Syndrome also play a significant role in disease presentation.

**Methods:**

This narrative review explores the dermatologic presentations of Hyper-IgE Syndrome in pediatric populations, including descriptions, associated symptoms/findings, and available treatment options.

**Results and conclusion:**

Neonatal rash, mucocutaneous candidiasis, noma neonatorum, psoriasis, cold staphylococcal abscesses, and candida onychomycosis are among the dermatological manifestations of Hyper-IgE Syndrome. Each manifestation has unique characteristics and treatment considerations, necessitating accurate recognition and diagnosis for effective management. Optimal treatment strategies involve a combination of supportive care, topical/systemic therapies, antifungal medications, and surgical interventions when necessary. Further research is needed to enhance our understanding of these manifestations and evaluate treatment modalities for individuals affected by Hyper-IgE Syndrome.

## Introduction

Hyper-immunoglobulin E syndrome (HIES), formerly known as Job’s syndrome, is an uncommon type of primary immunodeficiency disorder that impacts less than one person per million [1]. Cellular and humoral immune systems are both involved in this condition. Patients with HIES typically present with immune and nonimmune features [2]. Immune-related presentation include eczematoid dermatitis, recurrent skin infections, respiratory tract infections, mucocutaneous candidiasis, and elevated serum IgE levels [3]. Nonimmune features may manifest as high arched palate, prominent palatine ridges, reuretained primary teeth [4], and other skeletal abnormalities [3]. During adolescence, a distinct facial appearance often develops, characterized by facial asymmetry, deep-set eyes, a wide nose, and prominent skin pores [5, 6].

Diagnosing HIES can be challenging due to its clinical presentation, which may overlap with other conditions like chronic granulomatous disease, HIV infection, severe atopic dermatitis, or cystic fibrosis. Despite the high morbidity and mortality associated with AD-HIES, advancements in medical care, vigilant monitoring, and patient compliance have improved prognosis, allowing survival for 50 years or more. However, complications include an increased risk of malignancies such as non-Hodgkin’s lymphoma, Hodgkin’s lymphoma, vulvar and lung cancers, as well as autoimmune diseases like systemic lupus erythematosus, membranoproliferative glomerulonephritis, vasculitis, and dermatomyositis. Vascular abnormalities may lead to hypertension, while coronary and cerebral aneurysm ruptures can result in myocardial infarction and lacunar infarcts, respectively [7].

Cutaneous manifestations are often an early sign of the disease. The dermatological features of hyper IgE syndrome typically manifest as a rash on the scalp and face within the initial few weeks after birth, and biopsy may show eosinophilic infiltrations [8, 9]. Early diagnosis of HIES, especially the autosomal dominant form, can be challenging for dermatologists due to the lack of specific clinical features that may vary with age. This is further complicated because patients with severe atopic dermatitis may also exhibit high levels of serum IgE. In contrast, individuals with autosomal dominant HIES may initially present with moderate or normal serum IgE levels, making it more difficult to differentiate from other conditions, solely based on IgE levels in the early stages of the disease. Therefore, a comprehensive evaluation considering clinical symptoms, family history, and additional laboratory tests may be necessary to diagnose HIES accurately [10]. Besides, consultation with dermatologists are critical in identifying and distinguishing these manifestations from other dermatologic conditions with similar clinical features.

Understanding the dermatologic presentation of hyper IgE syndrome is crucial for accurate diagnosis and effective management of the disease. This narrative review provides a comprehensive overview of the various dermatological manifestations observed in the pediatric population with HIES, including descriptions, associated symptoms/findings, and available treatment options. This article aims to highlight the importance of recognizing dermatologic features in diagnosing Hyper-IgE Syndrome (HIES) in the pediatric population. By focusing on these dermatologic signs, this article provides guidance for dermatologists and other healthcare professionals on identifying HIES in children.

## Method

In order to collect relevant literature, a search strategy was employed in order to conduct this narrative review on the dermatologic presentations of HIES in pediatric populations. This review was conducted using PubMed as the primary database. Our search strategy aimed to identify articles that addressed dermatological manifestations of HIES in children.

The following keywords and Boolean operators were used to refine the search:

(((paediatric) OR (pediatric) OR (child*)) AND (“hyper IgE syndrome” OR “HIES” OR “IL6R” OR “IL6ST” OR “ZNF341” OR “SPINK5” OR “PGM3” OR “CARD11” OR “TGFBR1” OR “TGFBR2” OR “BCL11B” OR “ERBB2IP”)) AND ((dermato*) OR (cutaneous) OR (skin)) Utilizing these terms in combination with appropriate Boolean operators, we aimed to retrieve articles that were as relevant as possible. In addition, only articles published in English were included in the search. The inclusion criteria encompassed studies, case reports, and reviews that provided valuable insights into the dermatologic aspects of HIES in the pediatric population. Articles that did not meet these criteria were excluded. In order to ensure inclusion of the most recent and relevant literature concerning the dermatologic presentation of HIES in pediatrics, the search was conducted until February 1st, 2023. The obtained articles were then meticulously reviewed, and relevant information about the dermatological presentation of HIES in pediatric patients was extracted for the narrative synthesis.

## Genetic variants of hyper IgE syndrome

HIES can arise from pathogenic variants in various genes, including *IL6R*, *IL6ST*, *ZNF341*, *SPINK5*, *PGM3*, *CARD11*, *TGFBR1*, *TGFBR2*, *BCL11B*, and *ERBB2IP* [11].

Even though most cases of HIES are sporadic, it can also be inherited in an autosomal dominant (AD) or autosomal recessive (AR) manner [12]. AD-HIES, an autosomal dominant form of hyper-IgE syndrome, is caused by mutations in the signal transducer and activator of transcription 3 (*STAT3)* gene. This gene is found on chromosome 17 (Phenotype MIM Number = 147060), and these mutations have a dominant negative effect on the STAT3 protein. These mutations lead to impaired immune cell function and compromise immune surveillance [13]. The presentation of AD-HIES is characterized by a range of symptoms. These include eczematoid rashes, skin abscesses, recurrent sinopulmonary infections, and mucocutaneous candidiasis. Additionally, individuals with AD-HIES have an elevated risk of developing certain malignancies, particularly non-Hodgkin’s lymphoma (NHL). In most cases, the NHL associated with AD-HIES is characterized by aggressive histology and originates from B cells [14, 15, 16]. Over 140 different versions of STAT3 with two different alleles have been identified so far [17]. These variants have been found to cause disease in at least 95% of the tested alleles [17]. STAT3 is a transcription factor that is involved in various signaling pathways triggered by cytokines or growth factors. The complexity of these signaling pathways has made it challenging to comprehend how these STAT3 variants contribute to the development of HIES [18]. The identification of specific genetic mutations in the IL6ST gene, which codes for the GP130 protein, has shed light on the importance of the IL-6 cytokine family in the development of AR-HIES and AD-HIES. These mutations include biallelic partial loss-of-function (LOF) variants in AR HIES patients and monoallelic DN variants in AD HIES patients. This discovery emphasizes the significant role played by the IL-6 cytokine family in the underlying mechanisms of this disease [19, 20, 21]. In 2018, two studies revealed a novel genetic defect associated with autosomal recessive HIES. This defect involves biallelic loss-of-function (LOF) variations in the ZNF341 gene, which codes for an unknown protein. ZNF341 acts as a transcription factor, regulating the expression of STAT3 in both normal and inducible conditions [21, 22]. A recent 2023 study identified two additional patients with AR HIES and complete ZNF341 deficiency, further highlighting the crucial role of ZNF341 in the development of this syndrome [23]. When genetic testing is not available, autosomal recessive (AR) ZNF341 deficiency closely resembles autosomal dominant (AD) STAT3 deficiency in terms of clinical symptoms, although it tends to be less severe. ZNF341 and STAT3 deficiencies share many biological characteristics, except for reduced counts of natural killer (NK) cells and the presence of appropriate signs of inflammation in patients with ZNF341 deficiency [23]. CARD11 variant has been associated with HIES syndrome, mutations leading to gain of function effect, contributing to the symptoms. Given the predominant association of AD-HIES with STAT3 deficiency, it is more precise to refer to STAT3 when describing this specific syndrome [17].

In contrast, AR-HIES is associated with a higher susceptibility to viral cutaneous infections and more severe atopic eczema [24].

Initially recognized as the AR-HIES the autosomal recessive form of hyper-IgE syndrome, is caused by gene mutations in the DOCK8 gene. DOCK8 is located on chromosome 9 (MIM = 243700) [24], Dedicator of cytokinesis protein 8 (*DOCK8)* deficiency is currently classified as a combined immunodeficiency. While it is typically less severe than severe combined immunodeficiency (SCID), it does share certain clinical features with *STAT3* deficiency [25, 26] This further complicates the process of distinguishing HIES from other inborn errors of immunity (IEI).In particular, DOCK8 deficiency is associated with a unique presentation that includes severe cutaneous viral infections such as warts and Molluscum. Additionally, *DOCK8* mutations confer an increased risk of malignancy, which can occur at a younger age [24]. The 2019 study by Aydin SE et al. emphasized the significance of diagnosing patients with *DOCK8* deficiency, as it confirmed that hematopoietic stem cell transplantation (HSCT) is the sole curative treatment for this type of immunodeficiency [27]. Although *DOCK8* deficiency and *STAT3* deficiency are not classified in the same category, they share several clinical features commonly seen in HIES, including *STAT3* deficiency [28]. Distinguishing between the two based solely on clinical features can sometimes be challenging. For example, in a specific case, two patients exhibited clinical features that did not align with the typical phenotype of *DOCK8* deficiency, leading to initial suspicion of *STAT3* deficiency. This suspicion was supported by the observation of impaired STAT3 phosphorylation in these patients. However, the use of whole exome sequencing (WES) enabled a correction in the diagnosis, revealing that these patients actually had *DOCK8* deficiency [28].

Two newly identified genetic causes of HIES have been classified within the framework of Human Inborn Errors of Immunity (IEI). These novel entities are *ADIL6ST* partial deficiency and *ARIL6ST* complete deficiency [29].

Distinguishing between AR-HIES and AD-HIES solely based on clinical symptoms can be challenging because the symptoms may change over time. However, since the prognosis and treatment for AR-HIES and AD-HIES differ, it is crucial to differentiate between the two genetic types [27, 30].

## General clinical features

Studies have shown that recurring skin abscesses and pneumonia are the most common clinical symptoms observed in individuals with HIES [1, 30, 31].

Cranio-facial and dental abnormalities are common, with prominent forehead, interalar distance enlargement, and ear or nose soft tissue thickening. Retention of deciduous teeth is also seen in the majority of patients over the age of 8 years [30]. Abnormal bone fractures, joint hyperextensibility, unusual joint dislocation, and scoliosis are common skeletal features of HIES. Neurological manifestations, such as syringomyelia and posterior fossa arachnoid cysts, have also been reported. Cardiovascular manifestations include hypertension with left ventricular hypertrophy, anoxic brain hemorrhagic necrosis, deep venous thrombosis, leg phlebitis, and pulmonary embolism. Allergies, asthma, and skin manifestations may also be present. These varied clinical features make the diagnosis of HIES challenging and require a high index of suspicion for early diagnosis and treatment.

## Dermatological manifestations in the pediatric population

### Neonatal rash

Most patients with HIES have a previous occurrence of a rash that appears in newborns, which typically starts during the first month after birth [8]. About 67% of these patients develop a rash consisting of papules and pustules before they turn two months old, and around 71% of them develop a rash within two weeks after birth [10].

The histological examination of the affected skin in patients with HIES reveals the presence of eosinophilic spongiotic dermatitis, eosinophilic folliculitis, and perivascular dermatitis with a significant number of eosinophils observed in the superficial and deep layers [9].

Initially, many of these patients with HIES were misdiagnosed with neonatal acne because the papules and pustules started on the face and scalp, similar to neonatal acne. However, unlike neonatal acne, the rash gradually affects the entire upper body [8]. Neonatal acne is a term that is commonly used interchangeably with neonatal cephalic pustulosis [32]. It is essential to differentiate between HIES rash and neonatal acne because of the different courses of the rash. Sometimes, the rash associated with HIES develops into eczema and can have a prolonged course [32]. However, in some patients with HIES rash, treatment with oral antibiotics or topical corticosteroids can improve or resolve the rash [13].

The appearance of the rash and its distribution on the skin of newborns with HIES is also comparable to a skin condition known as eosinophilic pustular folliculitis of infancy [32]. The onset is usually older in eosinophilic pustular folliculitis, with most cases occurring between ages 5 and 10 months rather than in the first month [33]. It does not usually improve with antibiotics. However, it does improve with topical steroid treatment [34].

Other conditions, such as transient pustular melanosis and erythema toxicum neonatorum, should also be considered regarding pustules during the newborn period [32].

Compared to HIES, the pustules observed in transient pustular melanosis are typically more shallow and soft and usually disappear with the formation of erythematous macules [35].

The appearance, onset age, distribution, and histological features of erythema toxicum neonatorum can resemble the newborn rash observed in HIES. It typically manifests within the initial two days of life and resolves within five days. In some cases, mild reoccurrences may occur within 5 to 10 days after the initial outbreak [36]. Similar to the newborn rash of HIES, erythema toxicum neonatorum can be present at birth [36].

**Fig. 1:**
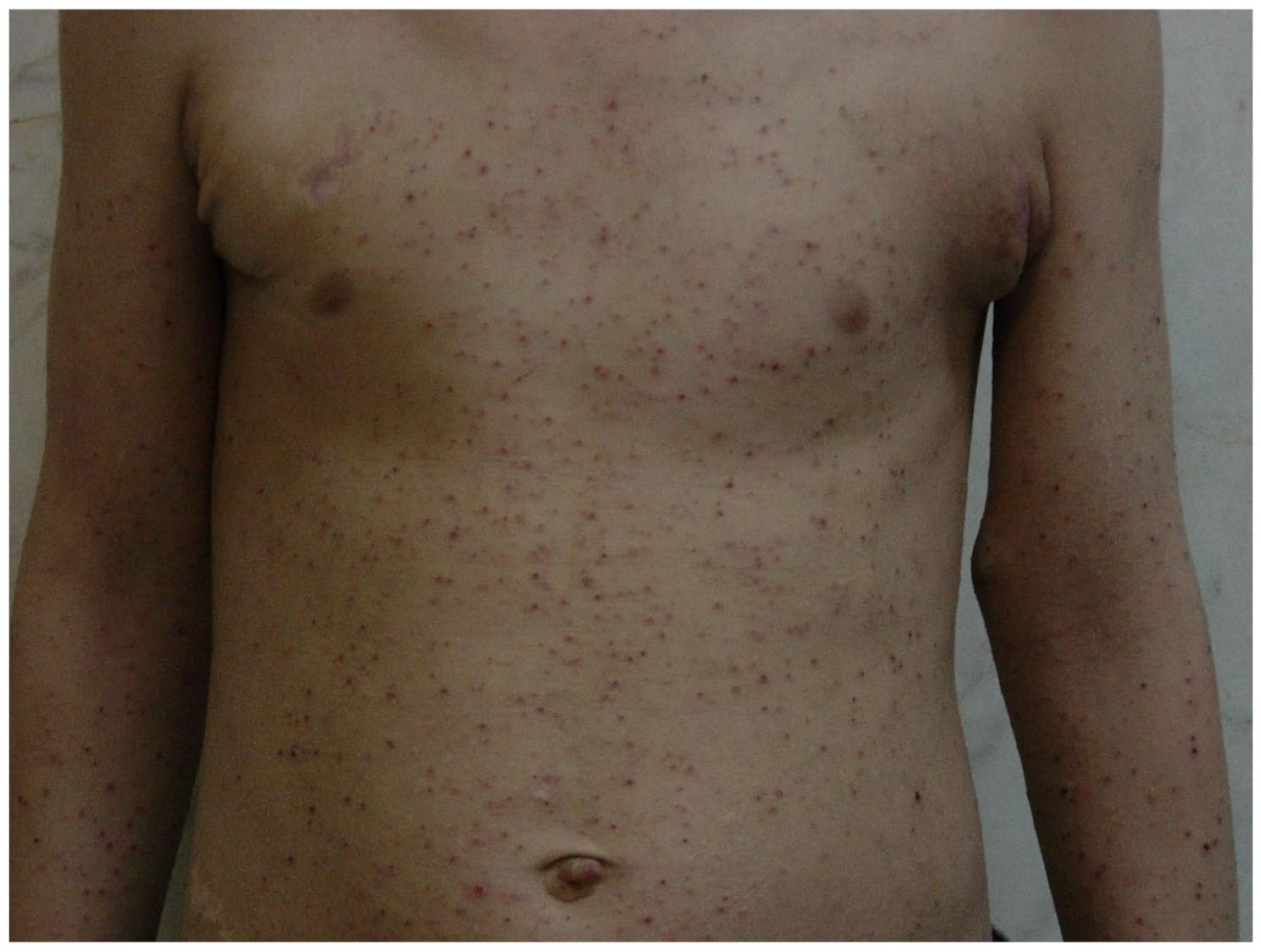
Generalized erythematous and excoriated papules suggesting an eczematous dermatitis in a 10-year-old boy with HIES

Most people with HIES commonly experience symptoms within the spectrum of a papulopustular rash and eczematous dermatitis (Fig. 1) [10]. Research indicates that approximately 65% of individuals diagnosed with HIES fulfill the criteria for atopic dermatitis, a common form of eczema. Furthermore, HIES and atopic dermatitis typically involve the presence of itching (pruritus) as a shared symptom [8, 9]. Individuals affected by HIES typically do not exhibit additional symptoms, which are commonly associated with allergies, such as hay fever or asthma. Moreover, they usually do not have a family history of atopy, defined as a predisposition to allergic conditions [37].

The neonatal rash is a critical symptom that appears in most individuals with HIES. Given that the distribution, appearance, and histologic characteristics of the neonatal rash in HIES are similar to other skin conditions, it is crucial to differentiate HIES from similar conditions for an accurate diagnosis and appropriate treatment. Therefore, healthcare professionals should be aware of the distinct features of neonatal rash in HIES to provide adequate care for affected individuals.

### Mucocutaneous candidiasis

Candida species, particularly Candida albicans, are fungi that commonly inhabit the skin and mucous membranes of the gastrointestinal and genitourinary tracts in a significant portion (around 30–50%) of healthy individuals within a given population at any given time. Most individuals experience colonization by Candida at some point in their lives. The human body’s natural defense mechanisms normally prevent this fungus from causing illness; however, if there is a breakdown in the skin or mucosal barriers, imbalances in the microbiome, or defects in the immune system, the risk of developing severe candidiasis, either on the skin or mucous membranes, or invasive candidiasis increases [38].

Superficial candidiasis which affects the mucous membranes and skin is very common. It can occur in individuals with weakened immune systems and those with a healthy immune system. In most cases, these Candida infections that affect the skin, nails, oropharyngeal mucosa, esophagus, and genital tract are sporadic and not severe [39].

Approximately 60% of individuals with HIES suffer from chronic mucocutaneous candidiasis, characterized by recurring fungal infections in the mucous membranes and skin. This symptom can be effectively controlled by employing a combination of antifungal medications administered orally and topically [10].

A significant reduction in the production of Th17 cells has been consistently observed in patients diagnosed with HIES [40, 41, 42]. More recent reports have highlighted that individuals experiencing recurrent mucocutaneous candidiasis, accompanied by decreased Th17 cell production, can be attributed to *CARD9* [32] and *DECTIN1* [33] gene mutations.

### Psoriasis

Psoriasis is uncommon in pediatric patients with HIES. While the association between psoriasis and HIES has been rarely reported, it is believed that the autoimmune nature of psoriasis can lead to its manifestation in individuals with immunodeficiency diseases [43].

The immune microenvironment in HIES lesions appears to be disrupted, leading to an imbalance of Th1/Th2 cytokines [6]. This imbalance can have effects beyond eczema dermatitis and may contribute to the development of additional changes. Psoriasiform lesions observed in HIES patients could be attributed to this dysregulation. Additionally, the recurrent infections commonly experienced by individuals with HIES can also be a potential contributing factor to the occurrence of psoriasiform lesions in these patients [43]. Further research is needed to fully understand the underlying mechanisms and relationship between immune dysregulation, recurrent infections, and the development of psoriasiform lesions in HIES.

There have been two reported cases of psoriasis and psoriasiform lesions occurring in patients with HIES. The first case was a 17-year-old boy with a history of recurrent skin inflammation and boils, who presented with severe and persistent erythroderma. The skin biopsy findings indicated thickened epidermis, increased production of skin cells, and the presence of neutrophils, Munro’s microabscesses, and immune cell infiltration, resembling the histological features of psoriasis [43].

The second case was a 16-year-old Iranian boy, who presented with a one-year history of white scaling papules on his knees and elbows, which were diagnosed as psoriasis based on a skin biopsy. Laboratory tests did not identify any genetic or molecular defects. Treatment included inhaled corticosteroids, bronchodilators, antibiotic prophylaxis, and occasional intravenous immune globulin (IVIG). The biopsy showed hyperkeratosis, parakeratosis, and elongated rete ridges in the epidermis, confirming the psoriasis diagnosis [44].

In conclusion, psoriasis is a noteworthy manifestation in the pediatric population with HIES, albeit a rare occurrence. Additional studies are needed to understand the connection between psoriasis and HIES in order to shed more light on the underlying mechanisms and potential treatment approaches for this unique patient population.

### Abscesses

Cold abscess in considered an important clue for diagnosis of HIES. Cold abscesses can often be mistaken for cysts or benign tumors. Although these abscesses may not show typical signs of inflammation, they can be large and are usually caused by S. aureus [45]. Fortunately, the incidence of cold abscesses in HIES patients has decreased due to early administration of antibiotics and prophylactic anti-staphylococcal antibiotics [32].

Patients with HIES have been found to have diminished acute-phase responses, such as decreased serum levels of C-reactive protein during infections. This could be due to defective signaling of IL-6, as the protein STAT3, which is involved in IL-6 signaling, is important in producing acute-phase proteins. This phenomenon’s exact molecular mechanism has yet to be fully understood [46]. However, it is known that HIES patients frequently experience recurrent skin infections with S. aureus, which can develop “cold” abscesses that lack typical signs of inflammation, such as warmth and redness. This is a nearly universal feature of HIES [32, 47].

Healthcare providers need to be aware of HIES symptoms and consider this syndrome in cases of recurrent abscesses.

### Cutaneous fusariosis

Fusarium species are molds found everywhere, which can cause infections in humans, but the risk of infection depends on the person’s immune system. Fusarium infection is usually limited to certain areas, such as the cornea, nails, and skin in people with a healthy immune system.

However, in patients with severe neutropenia, exposure to corticosteroids, or those who have had a hematopoietic stem cell transplant, even minor skin lesions can lead to the spread of the fungus throughout the body, known as secondary disseminated Fusariosis. This can cause necrotic skin lesions and fungemia (the presence of fungus in the blood), which are associated with high mortality rates [48, 49, 50].

A recent report discussed a rare condition known as primary invasive cutaneous Fusariosis, which was observed in four patients with a specific genetic mutation called *STAT3* dominant negative (DN). The infection primarily affected the extremities and was characterized by long-term involvement confined to the skin and subcutaneous layers, without spreading to other parts of the body. In all patients, Fusarium solani, a type of fungus, was identified through positive skin fungal cultures. Furthermore, one patient exhibited an eosinophilic inflammatory infiltrate in the deep subcutis [51]. The tissue abnormalities and defects in the immunological skin barrier found in *STAT3* DN patients may explain the development of Fusarium skin disease [30]. Research has indicated the essential role of *STAT3* in skin remodeling and maintaining its balance. In patients with *STAT3* DN mutations, there is an impaired expression of IL-17 and reduced differentiation of Th17 cells. This leads to an increased susceptibility to epithelial infections and an imbalance in the skin microbiota [52, 53].

The clinical presentation and treatment response vary among the patients, underscoring the importance of utilizing multiple therapeutic approaches, such as topical antifungal medications. These case reports offer valuable knowledge about the underlying mechanisms and treatment of primary invasive cutaneous Fusariosis in individuals with *STAT3* DN mutations. The findings emphasize the need to consider primary invasive cutaneous Fusariosis as a potential diagnosis in patients with persistent skin lesions and underlying immune deficiencies, particularly those associated with *STAT3* DN mutations. Further research is required to comprehensively understand the range of cutaneous fungal infections in immunocompromised patients and to develop effective treatment strategies.

### Lesions similar to atopic dermatitis

Atopic dermatitis, also called atopic eczema, is a prevalent chronic inflammatory condition that affects many people in wealthy countries, and approximately 20% of children [54]. It causes itching, dry skin, and eczematoid skin lesions [55, 56].

**Table 1:** A comparison between atopic dermatitis and hyper IgE syndrome in the pediatric population

	Atopic Dermatitis	Hyper-IgE Syndrome
Prevalence	Common in wealthy countries [54]	Less common [1]
Skin Lesions	Eczematoid, dry, itchy [55, 56]	Resemble atopic dermatitis with some atypical features [57]
Genetic Factors	*FLG* gene mutations [58, 59]	STAT3 or DOCK8 deficiency [12]
Skin Barrier	Compromised [58]	Impaired skin remodeling [32]
Distribution of Lesions	Varies with age [60]	Face and extensor surfaces [61]
Allergic Disorders	Linked to food allergy, asthma, and allergic rhinitis [62]	Less common, but can be present in DOCK8 deficiency [62]
Infections	Superficial staphylococcal infections [61]	Deep-seated abscesses, Mucocutaneous candidiasis [61]
Age of Onset	After 2–4 months of age [61]	Before 1 month of age [61]
Serum IgE Levels	High [61]	High [61]
Eosinophilia	Present [61]	Present [61]

A combination of genetic, immunological, and environmental factors causes atopic dermatitis. The exact cause is not yet fully understood, but it leads to a compromised skin barrier, allowing allergens, irritants, and microorganisms to enter the skin and trigger an immune response. Several genetic mechanisms have been identified as contributing to the risk of developing atopic dermatitis, with loss-of-function mutations in the *FLG* gene being the most consistently reported variants. This highlights the importance of the skin barrier, as the filaggrin protein found in the epidermis plays a significant role in its structure and function [58, 59]. Filaggrin is a protein found in the granular layers of the epidermis, and during the process of keratinocyte differentiation, it is broken down into several identical filaggrin molecules. These molecules then combine to create a strong protein-lipid matrix that protects the skin by preventing water loss and blocking the entry of harmless allergens and infectious microorganisms [32].

Eczematous lesions, intense itching, and a chronic or relapsing disease course characterize atopic dermatitis. The distribution of eczematous lesions varies with age. Infants often have acute lesions that appear as poorly defined redness, swelling, blisters, and clear fluid oozing. These lesions can be widespread but typically affect the face, cheeks, and trunk and do not usually involve the diaper area. In childhood (starting at age 2), eczema becomes more localized and chronic, with paler redness, dry skin, and poorly defined lesions that often affect the flexor surfaces and may thicken (lichenification) in chronic areas [60].

Various studies have reported different descriptions of the skin manifestations in HIES. For instance, one study described them as “infected eczematoid skin lesions [63], while others characterized them as a type of dermatitis that resembled atopic dermatitis but had some atypical features [57, 30].

Despite these varying descriptions, all previous studies have consistently reported that almost all.

HIES patients display skin manifestations that resemble atopic dermatitis or eczema [57, 63, 64, 65].

Skin manifestations of both HIES and atopic dermatitis are characterized by high serum IgE levels and eosinophilia. In HIES, the rash is typically papular and itchy and often exhibits lichenification, a thickening of the skin resulting from hypertrophy of the epidermis. The clinical and histopathological features of HIES rash are very similar to those of atopic dermatitis, but there are reported differences in the distribution of the rash. In atopic dermatitis, the rash typically affects the flexural surfaces of the body, while in HIES, it is located on the face and extensor surfaces. However, the location of atopic dermatitis can change with age, so it is unclear, whether or not the previously reported differences are due to an essential difference between the two conditions [61].

Atopic dermatitis is frequently linked to other allergic disorders, including food allergy, asthma, and allergic rhinitis, which are not typically present in most cases of HIES, particularly those involving *STAT3* deficiency [62].

Staphylococcal infections are usually superficial in atopic dermatitis, while HIES patients develop deep-seated abscesses. Mucocutaneous candidiasis is also a common complication of HIES but not atopic dermatitis. The onset of skin lesions in atopic dermatitis usually occurs after two to four months of age, while symptoms of HIES typically present before one month [61].

Skin abnormalities in hyper-IgE syndrome (HIES) extend beyond skin lesions resembling atopic dermatitis. Staphylococcus aureus colonization frequently takes place, leading to more severe dermatitis when infection sets in. Although acute episodes can be managed with treatment, recurrent episodes are likely to happen unless prophylactic antibiotics are taken. In addition, many HIES patients exhibit a rough texture in their facial skin, which is thick and doughy, despite having no history of severe acne. The underlying cause of this cutaneous manifestation is not yet fully understood, but it may be related to impaired skin remodeling [32].

A comparison between Atopic Dermatitis and hyper IgE syndrome in the Pediatric Population is provided in Table 1.

### Squamous cell carcinoma

Squamous cell carcinoma is a type of cancer, originating from keratinocytes, the cells found in the epidermis or squamous mucosal epithelium. It is a malignant tumor that can spread to other body parts if left untreated [66]. Squamous cell carcinoma commonly develops in areas of the skin that have experienced chronic irritation, such as burns or scar tissues, long-standing non-healing wounds, exposure to x-rays, or contact with certain chemical substances like arsenic and petroleum. Factors like chronic and recurrent infections and prolonged suppression of the immune system have also been identified as potential risk factors for the development of squamous cell carcinoma [66].

Recent research has highlighted the crucial role of the immune system in cancer prevention through a process called the immune surveillance [67]. Immunosuppressed individuals, including those with HIES, are reported to have a higher incidence of SCC [68].

Pediatric cases of head and neck carcinoma are infrequent occurrences. If a chronic ulcer displays signs of excessive growth beyond what is normally observed during the healing process, it should raise concerns about potential malignant transformation [66].

HIES patients face an elevated risk of developing malignancies, including lymphomas. Furthermore, patients with *DOCK8* deficiency, a subtype of HIES, have shown susceptibility to papillomavirus-induced SCC and lymphomas [69].

The clinical presentation of SCC in HIES patients can vary, with symptoms often manifesting as chronic ulceration or non-healing wounds demonstrating atypical proliferation. The choice of treatment modality depends on several factors, including tumor differentiation, metastasis, size, shape, location, and predisposing factors. Surgical interventions, such as excision surgery, Mohs surgery, cryosurgery, electrosurgery, and radiation therapy, are among the available treatment options [70].

To illustrate the complexities of SCC in HIES, a reported case of a 17-year-old patient with a history of eczema, recurrent middle ear infections, and a family history of leukemia is worth mentioning. The patient demonstrated multiple clinical manifestations of HIES, including immune deficiency, eosinophilia, and elevated IgE levels. Over several years, the patient experienced recurrent otitis media, purulent ear discharge, and subsequent complications, leading to the diagnosis of SCC originating from the external auditory canal [69]. The case underscores the importance of comprehensive management and regular follow-ups in HIES patients.

The association between HIES and skin malignancies, particularly squamous cell carcinoma, highlights the complex interplay between immunodeficiency, chronic infections, and genetic predisposition. Early diagnosis, active surveillance, and appropriate treatment strategies are crucial in managing SCC in HIES patients [68]. Further research is needed to unravel the underlying mechanisms and develop targeted therapeutic approaches to improve outcomes for individuals with HIES and associated skin malignancies. Please see Table 2 for a brief summary and comparison of dermatologic presentations of hyper IgE syndrome in children.

**Table 2:** A comparison of dermatologic presentation of hyper IgE syndrome in the pediatric population

Dermatological Manifestation	Description	Associated Symptoms/Findings	Treatment
Neonatal rash	Skin rash that appears in newborns typically during the first month after birth [8] Gradually progresses to affect the entire upper body [8]	Red, blotchy patches or small bumps on the skin [8]	Oral antibiotics or topical corticosteroids can improve or resolve the rash [13]
Mucocutaneous Candidiasis	Fungal infection affecting the skin, nails, and mucous membranes [39]	Red, itchy rash with satellite lesions in the affected areas [38] Associated with decreased Th17 cell production [40]	Antifungal medications like fluconazole or clotrimazole [10]
Psoriasis	Chronic autoimmune condition causing rapid skin cell turnover [43]	Red, scaly patches on the skin, often with itching and pain [43] Autoimmune nature may contribute to its manifestation [43]	Topical corticosteroids, immunosuppressive drugs, and phototherapy [44]
Abscesses	Cold staphylococcal abscesses, often mistaken for cysts or benign tumors [32]	Red, swollen, and painful area with a palpable mass [32] Associated with decreased acute-phase responses and recurrent S. aureus infections [46]	Incision and drainage, antibiotics if necessary [32]
Cutaneous Fusariosis	Fungal infection caused by Fusarium species [49] Infection primarily affects the extremities in patients with STAT3 DN mutations [51]	Reddish-brown nodules or ulcers with surrounding erythema [49]	Antifungal medications, such as voriconazole or amphotericin B [51]
Lesions similar to atopic dermatitis	Skin lesions resembling atopic dermatitis [55]	Red, inflamed patches with scaling and itching [56]	Topical corticosteroids, moisturizers, avoidance of triggers [61]
Squamous cell carcinoma	Malignant skin cancer originating from squamous cells [66]	Firm, red nodules or flat, scaly patches that may bleed or ulcerate [66]	Surgical excision, radiation therapy, chemotherapy [70]

## Future directions

In dermatology, HIES presents unique challenges. It is crucial for clinicians to recognize the nuances of HIES, because of the variability in skin manifestations, often confused with more common conditions like eczema. A more precise diagnosis of this disease has been made possible by advances in genetic and immunological testing. However, early diagnosis without resorting to invasive tests remains a significant challenge.

In addition to reduced economic burden on healthcare systems, a deeper understanding of dermatological presentations may also lead to quicker diagnoses. The question remains: how can this knowledge be effectively applied in clinical practice? It might be tough to implement advanced diagnostic techniques because of a lack of standardized guidelines, training, and economics.

Differentiating HIES dermatological symptoms from other pediatric skin disorders also poses a challenge. Hyper IgE syndrome (HIES) can manifest in various ways, presenting with respiratory or skin infections. It is important to consider this syndrome when encountering rare or treatment-resistant skin disorders, especially those showing unusual manifestations.

In cases where common skin issues like atopic dermatitis are not present, investigating for primary immunodeficiency disorders, including HIES, is advised. Concerning atopic dermatitis (AD), its clinical features significantly overlap with various IEIs. Recognizing early signs of IEIs is critical as these conditions do not respond well to standard AD treatments. Unique features of IEIs may become apparent at birth or develop over time, necessitating early identification to prevent complications. Therefore, it is recommended to assess children with an atypical AD course for potential IEIs. Indicators of IEIs can be subtle, ranging from early-onset eczema before two months of age to various symptoms such as recurrent diarrhea, endocrinopathy, autoimmunity, viral skin infections, neoplasms, failure to thrive, food allergies, recurrent infections, or associated features like bamboo hair, developmental delay, and skeletal anomalies. A comprehensive work-up, including a complete blood count with lymphocyte subsets, serum levels of IgG, IgM, IgA, and IgE, and vaccination titers for diphtheria, tetanus, H. influenzae, and pneumococcus, is recommended for all suspected IEI cases. While this initial assessment can be conducted by any physician for a swift diagnosis, timely referral to a clinical immunologist is essential [71].

## Conclusion

Understanding the dermatologic presentation of HIES is crucial for accurate diagnosis and effective management of the disease. This review article provided an overview of HIES, a rare primary immunodeficiency disorder, focusing on its dermatologic manifestations in pediatric patients. The article aimed to highlight the importance of recognizing dermatologic features in diagnosing and managing HIES in the pediatric population and to provide guidance for dermatologists and other healthcare professionals caring for patients with HIES. Early diagnosis and management can help prevent or minimize the morbidity associated with HIES. Therefore, an interdisciplinary approach involving dermatologists, immunologists, and other specialists is essential for optimal care of patients with hyper IgE syndrome.

In conclusion, dermatological manifestations in the pediatric population can provide important clues for diagnosing and managing various conditions, including HIES and other immunodeficiency disorders. Neonatal rash is a common symptom in HIES, and distinguishing it from other skin conditions, such as neonatal acne, transient pustular melanosis, and erythema toxicum neonatorum, is crucial for accurate diagnosis and treatment. HIES is also associated with mucocutaneous candidiasis, characterized by recurring fungal infections in the mucous membranes and skin, which can be effectively controlled with antifungal medications. Additionally, psoriasis and psoriasiform lesions have been reported in a small number of pediatric patients with HIES, suggesting a possible connection between immune dysregulation and the development of these conditions. Cold staphylococcal abscesses are a nearly universal feature of HIES, and healthcare providers should be aware of this symptom when evaluating patients with recurrent abscesses. Candida onychomycosis, although rare in children, should prompt investigation for underlying immunosuppression, including HIES. Fusarium skin infections have been observed in patients with *STAT3* Dominant Negative mutations, highlighting the importance of maintaining the immunological skin barrier, juvenile dematomyositis have been reported in two cases of HIES associated with recurrent abcesses. Overall, understanding the dermatological manifestations in the pediatric population can aid in early diagnosis, appropriate management, and improved outcomes for patients with these conditions. Further research is needed to elucidate the underlying mechanisms and establish optimal treatment approaches for these dermatological manifestations in pediatric patients.
